# Egyptian cobra (*Naja haje haje*) venom phospholipase A2: a promising antiviral agent with potent virucidal activity against simian rotavirus and bovine coronavirus

**DOI:** 10.1007/s00203-022-03139-7

**Published:** 2022-07-27

**Authors:** Walaa H. Salama, Mohamed N. F. Shaheen, Yasser E. Shahein

**Affiliations:** 1 Molecular Biology Department, Biotechnology Research Institute, National Research Centre, Dokki, Cairo, 12622 Egypt; 2 Environmental Virology Laboratory, Water Pollution Research Department, Environment and Climate Change Research Institute, National Research Centre, Dokki, Cairo, 12622 Egypt

**Keywords:** Cobra, Venom, Rotavirus, COVID-19, Phospholipase A2, Antiviral activity

## Abstract

Viral infections are linked to a variety of human diseases. Despite the achievements made in drug and vaccine development, several viruses still lack preventive vaccines and efficient antiviral compounds. Thus, developing novel antiviral agents is of great concern, particularly the natural products that are promising candidates for such discoveries. In this study, we have purified an approximately 15 kDa basic phospholipase A2 (PLA2) enzyme from the Egyptian cobra *Naja haje haje* venom. The purified *N. haje* PLA2 showed a specific activity of 22 units/mg protein against 6 units/mg protein for the whole crude venom with 3.67-fold purification. The antiviral activity of purified *N. haje* PLA2 has been investigated in vitro against bovine coronavirus (BCoV) and simian rotavirus (RV SA-11). Our results showed that the CC_50_ of PLA2 were 33.6 and 29 µg/ml against MDBK and MA104 cell lines, respectively. Antiviral analysis of *N*. *haje* PLA2 showed an inhibition of BCoV and RV SA-11 infections with a therapeutic index equal to 33.6 and 16, respectively. Moreover, *N. haje* PLA2 decreased the BCoV and RV SA-11 titers by 4.25 log_10_ TCID_50_ and 2.5 log_10_ TCID_50_, respectively. Thus, this research suggests the potential antiviral activity of purified *N. haje* PLA2 against BCoV and RV SA-11 infections *in vitro*.

## Introduction

Rotaviruses (RV) and bovine coronaviruses (BCoV) are identified globally as the most important enteropathogens responsible for acute viral gastroenteritis leading to diarrhea in infants, young children and animal species. Both types of viruses are important in global health and also in animal industries. At present, no approved antiviral medications are effective in treating infections caused by the two viruses. Therefore, the recovery is primarily dependent on fluid and electrolyte replacement until the infection is resolved (Leung et al. 2005). The newly emerged COVID-19 disease caused by the SARS-CoV-2 virus, a virus belonging to the coronavirus family, first appeared in Wuhan and in a few months became a pandemic and disrupted the entire world (Zhou et al. 2020; Wu et al. 2020). Therefore, the utmost efforts have been made to overcome this pandemic, and new therapeutic and vaccination approaches have been approved by the WHO for emergency use. Despite this, COVID-19 pneumonia treatment remains challenging, as new variants are able to escape from the available vaccines. In addition, many non-specific antiviral drugs are prescribed to reduce the severity of COVID-19 symptoms, despite their insufficiently effective use in elderly patients. Various antiviral drugs as well as natural products are currently being investigated for COVID-19 treatment (Kim 2021). In addition, several evaluated viral inhibitors against the SARS-CoV-2 virus in vitro are being screened for their potential effects in animal models and patients, such as main protease inhibitors (Dampalla et al. 2021; Gunst et al. 2021).

Animal venoms are considered as a treasure of numerous active pharmacological proteins and peptides (El-Aziz et al. 2019). In particular, snake venom contains different enzymes and polypeptide toxins exhibiting various biological and pharmacological activities against bacteria, fungi, viruses and tumors (El-Hakim et al. 2011; Salama et al. 2018; Roy and Bharadvaja 2021). Nowadays, several drugs are ascribed to patients derived from snake venom such as Captopril, a strong hypotensive drug approved by the FDA, and was discovered from bradykinin potentiating peptide (BPP) purified from Brazilian *Bothrops jararaca* pit viper venom (Costa et al. 2018). Tirofiban (Aggrastat) and Eptifibatide (Integrilin) are two antithrombotic drugs synthesized from snake venom disintegrins and prescribed for the treatment of acute coronary disease and angina (Rashidi et al. 2020). In that way, many trails have been done to express snake venom proteins that exhibit therapeutic activities by different biotechnological strategies to increase the yield of these components. Russo et al. (2019) expressed two recombinant PLA2 isoforms of *Crotalus durissus terrificus* in a prokaryote system and the purified recombinants showed similar antiviral properties against enveloped viruses such as Zika virus (ZIKV), Chikungunya virus (CHIKV), Dengue virus (DENV-2) and Yellow fever virus (YFV) compared to native PLA2. Thus, the necessity of exploring venom and its components as potential alternative therapeutics against viral infections (Rivero et al. 2011; Koh and Kini 2012; da Mata et al. 2017; Abidin et al. 2019; Ghosh et al. 2019), particularly Rota and Corona viruses, is increasing.

Snake venom contains a variety of enzymes such as serine proteases, metalloproteases, acetylcholine esterases, L-amino acid oxidases, hyaluronidases and phospholipases A2 (PLA2). Currently, several studies have demonstrated the potential therapeutic properties of venom enzymes purified from various species of snakes, which are progressively acquiring an attractive interest in their use in the biomedical field (Cedro et al. 2018). Phospholipases A2 (PLA2) (EC: 3.1.1.4) are Ca^+2^-dependent enzymes with low molecular masses (14–18 kDa) that catalyze the hydrolysis of phospholipids at sn-2 position producing lysophospholipids and free fatty acids, particularly arachidonic polyunsaturated fatty acid. According to the classification of phospholipases based on their structure, catalytic action and localization, the elapids (tropical and subtropical snakes) and hydrophids (sea snakes) PLA2s belong to secreted PLA2 group IA. On the other hand, vipers and cortalids PLA2s belong to group IIA, except many sPLA2s in *Bitis* sp. are listed in group IIB (Six and Dennis 2000; Filkin et al. 2020).

Phospholipases A2 are mostly abundant in elapids, particularly *Naja sp.*, and display varied functional pharmacological effects such as hemolysis, edema, neurotoxicity, myotoxicity, cytotoxicity, anticoagulation, immune modulating, antibacterial and antitumor effects (Kang et al. 2011; Trento et al. 2019). Venom composition is relatively varied among species and within the same species. Age, diet and geographical distribution are factors that affect the abundance and presence of venom enzymes (Modahl et al. 2020). PLA2 content varies within the same subgenus (*Uraeus*); Moroccan cobra (*Naja haje legionis*) has lower PLA2 content (3–4%) compared to Nigerian *Naja haje* (26%) (Malih et al. 2014; Hempel et al. 2022; Adamude et al. 2021). The Senegal cobra, *Naja senegalens*, found in western Africa, on the other hand, lacks PLA2 activity (Wong et al. 2021). Furthermore, the proteomics of Malaysian and Thailand *N. kaouthia* cobras demonstrated a significant variation in PLA2 activity (Modahl et al. 2020). However, the proteome of the Egyptian cobra (*Naja haje haje*) is still unpublished.

The Egyptian cobra (*Naja haje haje*) belongs to *Elapidae* family and is located in the cultivated areas around the Nile, Delta and Western Mediterranean Coastal Desert. It is one of the most medically important snakes, and its envenomation causes severe neurotoxic and myotoxic symptoms and may lead to death. In a previous study, the crude *N. haje* venom and its fractions showed moderate virucidal properties against herpes simplex viruses type I and type II (HSV-I and HSV-II) (Elsayed et al. 2014). Thus, the aim of the current work is to investigate the antiviral effect of phospholipase A2 purified from the Egyptian Cobra, *Naja haje haje* (*N. haje*) on rotavirus gastroenteritis and mammalian coronavirus in vitro.

## Material and methods

### Snake venom and materials

*Naja haje* venom was milked from snakes obtained from the farm of the Egyptian company for production of vaccines, sera and drugs (VACSERA). The pooled venom was centrifuged at 10,000×*g* for 10 min at 4 ℃ to remove debris prior to lyophilization and storage at ‒ 20 ℃ until use. The chromatographic resins and substrates for the biochemical and enzymatic assays were purchased from GE Healthcare, Thermo Scientific and Sigma-Aldrich companies. The reagents were of analytical grade.

### Viruses and cell culture

The Madin-Darby bovine kidney (MDBK) and African Green monkey fetal kidney (MA104) cells were purchased from VACSERA. The Mebus strain of bovine coronavirus (BCoV) as a surrogate model for SARS-CoV-2 was kindly obtained from the Department of Virology, Faculty of Veterinary Medicine, Cairo University. A simian rotavirus SA-11 (RV SA-11) as a surrogate model for human rotavirus was obtained from the Department of Virology, National Institute for Cholera and Enteric Diseases (NICED), Kolkata, India. Cells were grown in Dulbecco’s Modified Eagle Medium (DMEM) supplemented with 10% of heat inactivated fetal bovine serum (FBS), 100 units/ml penicillin, 100 μg/ml streptomycin under 5% CO_2_ humidified incubator (all purchased from Lonza, Belgium).

### Purification of PLA2 enzyme from *Naja haje* venom (Nh-PLA2)

The PLA2 enzyme was purified from *N. haje* venom using two consecutive chromatographic steps on sephacryl S-200 molecular exclusion chromatography followed by CM-sepharose ion exchange chromatography. First, *N. haje* crude venom (120 mg) dissolved in 1 ml of 50 mM Tris–Hcl buffer, pH 7, was applied to a sephacryl S-200 column (1.6 × 90 cm) previously equilibrated with the dissolving buffer. The elution was carried out with the same buffer, collecting fractions of 3 ml/tube at a flow rate of 20 ml/h at room temperature. All fractions were monitored at 280 nm for protein, and phospholipolytic activity was determined at 900 nm using egg yolk suspension as a substrate according to the Marinetti (1965) method. The fractions showing phospholipolytic activity were pooled, stored for further purification and named the S-N.h.PLA2 fraction. For the second step, the active pool (25 mg) was applied to a CM-sepharose column (1 × 12 cm), previously equilibrated with 50 mM Tris–HCl buffer, pH 7, at room temperature. The unbound proteins were washed with the equilibration buffer. Next, fractions were eluted in a step-wise method using different molarities of KCl at 0.05, 0.1, 0.15, 0.3, 0.6 and 1 M dissolved in the same buffer, and then 4 ml fractions were collected at a flow rate of 1 ml/min. Protein and phospholipolytic activity were determined. The fractions with high PLA2 activity (named Nh-PLA2) were pooled, dialyzed against dist. water, lyophilized and stored at − 20 ℃ until used for further study.

### Molecular mass determination

SDS-PAGE of the purified PLA2 (Nh-PLA2) was performed according to Laemmli (1970) using 15% polyacrylamide gels under reducing conditions. The gel was stained using Coomassie brilliant blue R-250. The pre-stained protein ladder with known molecular mass bands was used as a standard marker, with 13 major protein bands resolved in the polyacrylamide gel.

### Gel zymography

The purified Nh-PLA2 was run under non-reducing conditions on either a 15% SDS-PAGE or a 7% IEF gel followed by protein transfer onto a nitrocellulose membrane according to the method by Towbin (1979). Blood and egg yolk zymography according to the Moreno (1988) method was used to determine the molecular weight and isoelectric point (pI) of PLA2. Briefly, the membrane was incubated for 2 h with 1% casein hydrolysate dissolved in 50 mM barbiturate buffer, pH 8.6. For zymography on blood and egg yolk, the nitrocellulose membrane was placed directly on a 1% agarose gel containing 4% washed human erythrocytes, 4% egg yolk and 10 mM CaCl_2_ dissolved in 50 mM PBS, pH 7.4. After overnight incubation at 37 ℃, the clear zone indicated the presence of PLA2.

### Isoelectric focusing

Isoelectric focusing gel were performed as described by Garfin (1990), using a 7% polyacrylamide gel containing carrier ampholytes with pH ranging from 3 to 10 (Pharmalyte, Sigma-Aldrich). The isoelectric focusing marker with known pIs is used as a standard. The gel was silver stained.

### Phospholipase activity

The phospholipolytic activity of the chromatographic fractions was determined turbidimetrically according to Marinetti (1965) method. Briefly, 0.1 ml of fractions was added to 1 ml of working egg yolk suspension diluted in saline to give a final volume of 5 ml. Saline (0.1 ml) was added to the assay mixture and used as a control. The control was adjusted to give an absorbency of 0.7 (at 900 nm). The reaction velocity was determined as the change in OD per 5 min at 900 nm. Additionally, to determine the PLA2 activity of the pool active fraction (Nh-PLA2), the diameter of the hemolytic halo was measured in mm using an egg yolk–erythrocytes–1% agarose plate as a substrate according to the Gutierrez (1988) method. Briefly, assessed samples were diluted in PBS and applied to the substrate gel, and PBS was used as a negative control. After an overnight incubation of the samples on the plate at 37 ℃, the PLA2 activity was expressed as the size (in mm) of the hemolytic halos formed by each sample. One PLA2 unit was defined as the protein concentration that induces a hemolytic halo of 1 cm in diameter.

### Protein quantification

Protein content was determined according to Bradford (1976) method, using bovine serum albumin (BSA) as a standard.

### Cytopathic effect

The BCoV and RV SA-11 stocks were diluted tenfold and inoculated onto MDBK and MA104 cells, respectively, and the cytopathic effect was assessed after 72 h of incubation. The 50% tissue culture infectious doses/0.1 ml (TCID_50_/0.1 ml) were estimated as described previously by the Karber (1931) method, then stored in small aliquots at ‒ 80 ℃ until used.

### Cytotoxicity assay

The cytotoxicity of the PLA2 was investigated using the MTT assay after 4 days of cell culture according to the previously described protocol by Abid et al. (2012). Briefly, 5 × 10^3^ cells/well were seeded in 96-well plates. After 24 h, the growth medium was removed and the cell monolayers were incubated with various PLA2 protein concentrations (30, 15, 7.5, 3.75, 1.875, 0.9375, 0.469, 0.2344, 0.1172 µg/ml). After an additional 48 h at 37 ℃ under a humidified 5% CO_2_ atmosphere, the PLA2 was discarded and 100 μl of MTT solution (5 mg/ml) was added to all wells. After 4 h at 37 ℃, the MTT was carefully removed from the wells and replaced with 50 μl of dimethyl sulfoxide (DMSO). The plates were further incubated for 30 min at 37 ℃. The optical density was read on a multi-well ELISA reader at 540 nm. The 50% cytotoxic concentration (CC_50_) was calculated as (A‒B/A) × 100, where A and B are equal to the mean of three OD_540_ of untreated and treated cells, respectively. All experiments were repeated three times and the results were expressed as a percentage of cell viability in comparison to the untreated control cells.

### Antiviral assay

One hundred microliters of BCoV at 10^6.5^ log_10_TCID_50_/0.1 ml or RV SA-11 at 10^6^ log_10_TCID_50_/0.1 ml were incubated separately with the same volume of three non-toxic concentrations (1.875, 3.75, and 7.5 µg/ml) of PLA2 for 1 h at 37 ℃. One hundred microliters of the above mixture was added to a sub-confluent monolayer cells. After 1 h of incubation, the mixed solution was removed. The cell lines were washed twice with PBS, and then incubated with 200 μl of FBS free DMEM. Cell control (test medium without samples) and virus control (the virus suspension) were included. All plates were incubated for 3 days at 37 ℃ in CO_2_ incubator or until typical CPE was visible. The 50% inhibitory concentration (IC_50_) was defined as the PLA2 protein concentration that can protect 50% of viable cells from the cytopathic effect caused by virus and it was calculated by the MTT method as stated above in the cytotoxicity assay by multi-well ELISA reader at 450 nm as [(A‒B)/(C‒B) × 100], where A, B and C indicate the mean three absorbance of the test protein with virus infected cells, positive virus control, and negative cell control, respectively. Then the therapeutic index (TI) was estimated as the ratio of CC_50_/IC_50_. For determination of the yield reduction assay, tenfold dilutions of the virus were prepared in FBS free growth medium. One hundred microliters of viral dilutions 10^–4^–10^–9^ was mixed and incubated with 100 μl of tested PLA2 at a concentration of 3.75 μg/ml for 1 h. Microscopic examination for CPE was performed after 3 days post-infection. The virus titer as 50% tissue culture infection dose (TCID_50_) was calculated using the method by Karber (1931). The reduction of virus titer was estimated as the difference between the values of virus with PLA2 against virus without PLA2.

### Statistical analysis

The results were expressed as Means ± S.D. (*n* = 3). Data were analyzed using the Student’s *t* test using GraphPad Prism 8.0 software.

## Results and discussion

**Fig. 1:**
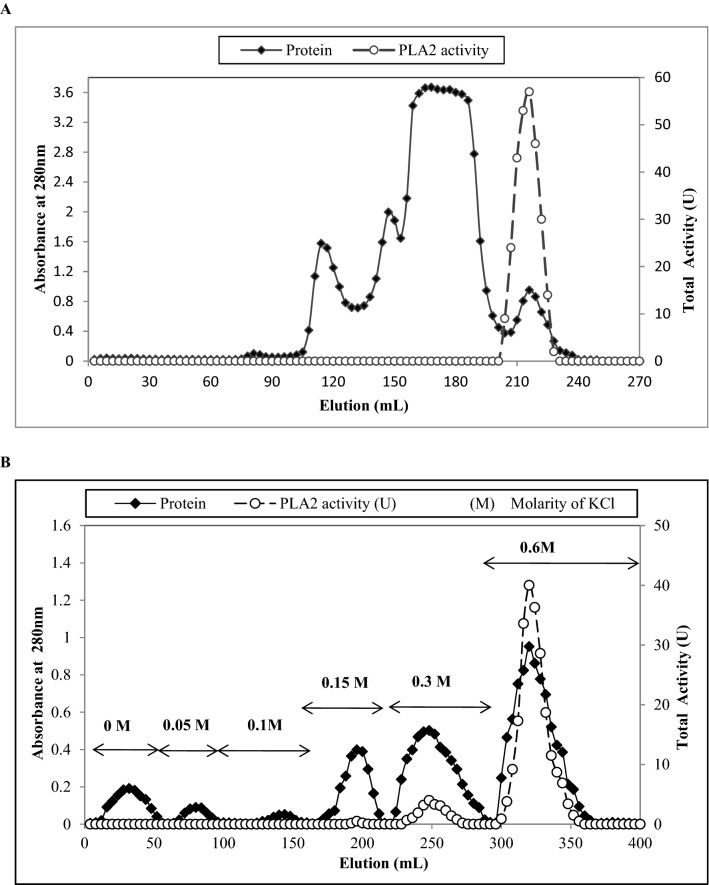
Purification of phospholipase A2 (PLA2) from *N. haje* venom in two chromatographic steps. **A** Gel filtration of 120 mg of *N. haje* dissolved in 1 ml of 0.05 M Tris–HCl buffer, pH 7 was applied to a Sephacryl S-200 column (1.6 × 90 cm) and, **B** Ion exchange chromatography of 25 mg of Active PLA2 pool on CM-Sepharose column (0.6 × 10 cm). The sample was equilibrated and washed with the dissolved buffer and eluted with different molarities of KCl (0.05, 0.1, 0.15, 0.3, 0.6, 1 M) in the same buffer. The column fractions were collected at a flow rate of 60 ml/h. The fractions with PLA2 activity were pooled, dialyzed, freeze-dried, and stored as Nh-PLA2

**Fig. 2:**
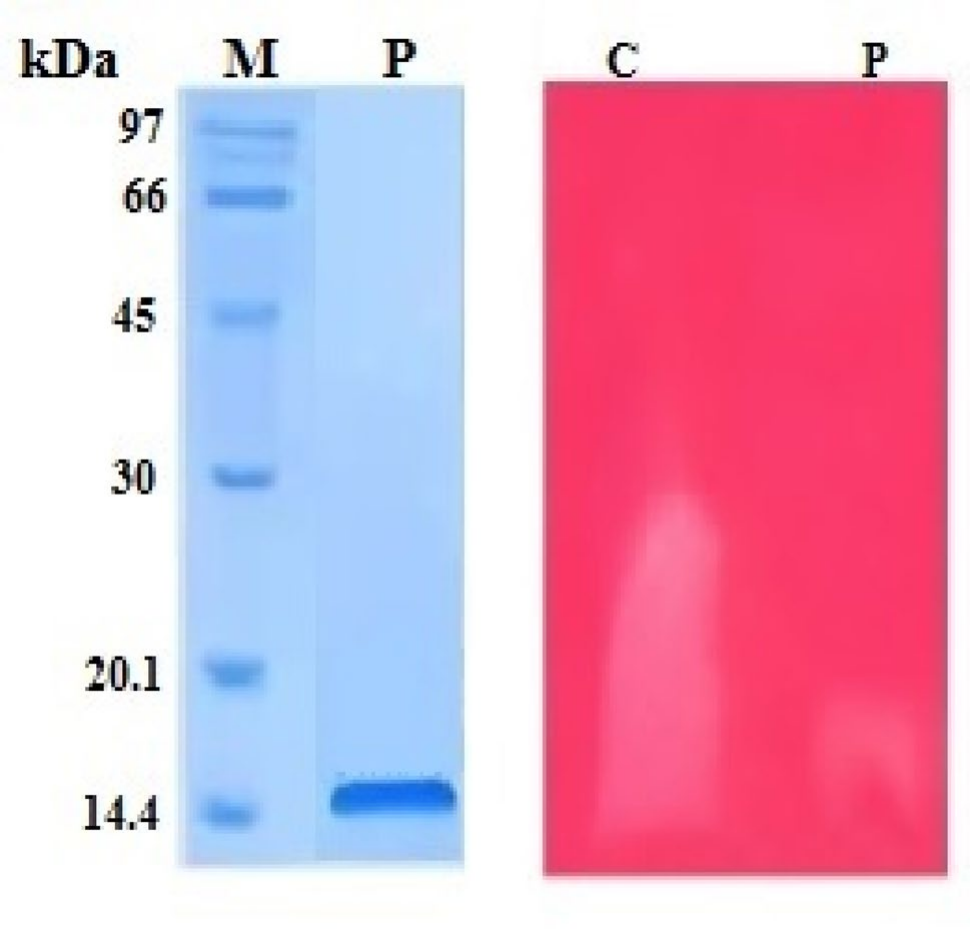
15% SDS-PAGE of the purified *N. haje* PLA2 (30 µg) under reducing conditions. The samples were: protein ladder ranging from 14.4 to 97 kDa (M), and purified *N. haje* PLA2 (P). After that, the purified PLA2 was electro-transferred onto nitrocellulose paper then incubated with agarose–RBCs–egg yolk substrate gel. The samples are *N. haje* venom (C), and *N. haje* PLA2 (P)

**Fig. 3:**
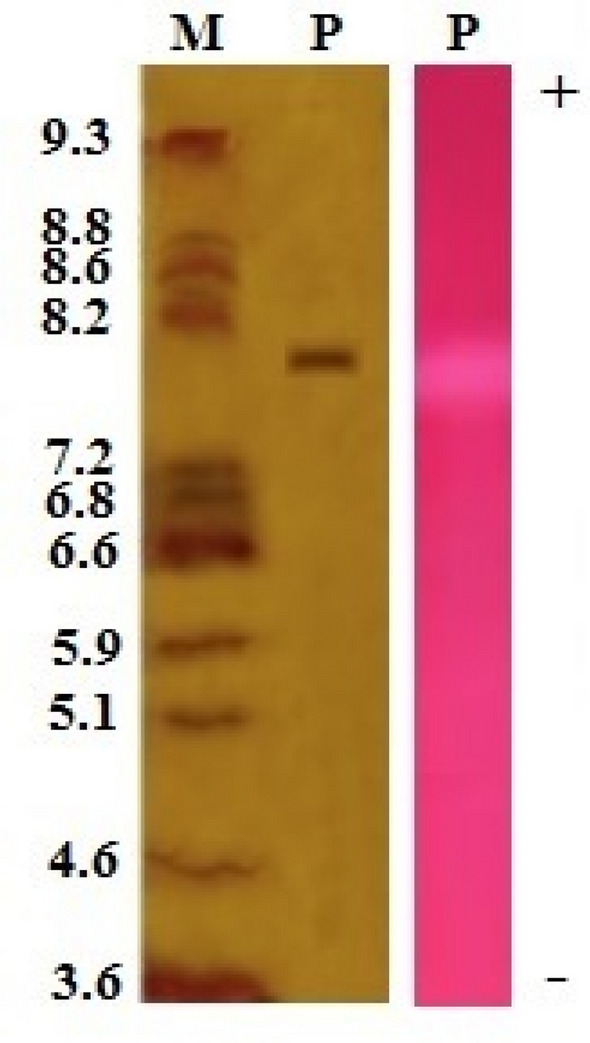
IEF gel (5%) of 10 µg of purified PLA2 (P) and IEF marker (M) under non-reducing conditions. After that, the purified PLA2 (P) was electro-transferred onto nitrocellulose membrane followed by incubation with fortified agarose gel. A hemolytic band was observed

Phospholipase A2 (PLA2) was purified from *N. haje* venom using gel filtration followed by cation exchange chromatography on sephacryl S-200 and CM-sepharose columns, respectively. First, four protein peaks were resolved from *N. haje* venom (120 mg) and phospholipase A2 activity was monitored in protein peak number 4 as shown in Fig. 1A. The active pool (S-N.h.PLA2) was 25 mg protein and total PLA2 activity of 275 U, respectively (Table 1). The active pool was applied to an equilibrated CM-sepharose column. Four major protein peaks were resolved as one unbound and three bound peaks were eluted at 0.15, 0.3, and 0.6 M of KCl dissolved in the equilibration buffer (Fig. 1B). The major PLA2 peak was eluted at 0.6 M KCl with a specific activity of 22 U/mg. The protein content and total activity of the purified PLA2 enzyme were 10 mg and 220 U, with 3.67-fold purification and 30.56% recovery (Table 1). The purified *N. haje* PLA2 had a molecular mass of approximately 15 kDa as determined by SDS-PAGE gel (Fig. 2). The molecular mass of the purified *N. haje* PLA2 was in agreement with the range generally reported for snake venom PLA2 (14–18 kDa) (Six and Dennis 2000). Furthermore, the molecular masses of the Egyptian *Naja nigricollis* and Saudi *Walterinnesia aegyptia* were approximately 14 kDa (Wahby et al. 2013; Abid et al. 2020). A hemolytic band of low molecular weight was resolved when PLA2 was loaded in 15% SDS-PAGE under non-reducing conditions and then placed on RBCs–egg yolk–agarose gel activity (Fig. 2). The pI of PLA2 enzyme was about 8, which confirmed by gel activity zymography showed a hemolytic band at the same point (Fig. 3). The assay of Moreno et al. (1988) was used to detect the enzymatic PLA2 activity based on the transfer of the resolved protein to a substrate-fortified gel. This assay was highly useful in identifying the molecular mass and the pI of the PLA2 enzymes present in biological fluids in general and snake venoms in particular.

**Table 1:** Purification of PLA2 enzyme from Egyptian *N. haje* snake venom

Sample	Total protein (mg)	Total activity (U)	Specific PLA2 activity (U/mg protein)	Recovery %	Fold purification
*N. haje* venom	120	720	6	100	1
S-N.h.PLA2	25	275	11	38.19	1.83
Nh-PLA2 (0.6 M)	10	220	22	30.56	3.67

Currently, specific antiviral agents and vaccines are still insufficient to prevent or control emerging and re-emerging viral illnesses (Kaufmann et al. 2014; Maslow 2018). Consequently, the development of novel antiviral agents is mandatory. In general, drugs targeting the virus itself or interrupting viral replication are the only approaches to specifically treat viral diseases (Mohammadi et al. 2019). Thus, a wide range of compounds extracted from natural sources has been used against various diseases (Brahmachari 2011). Of these compounds, snake venom is the source of many drugs that have been licensed by the FDA or are in preclinical or clinical trials for a variety of medicinal purposes (Koh and Kini 2012; Calderon et al. 2014; Abd El-Aziz et al. 2019 et al. 2019). Due to the variety of venomous snake species as well as venom composition, several studies have been done to evaluate the potential antiviral activity of both whole venom and its components against different types of viruses. sPLA2s and proteases are major enzymes present in elapids, vipers and crotalids. Most elapids have higher PLA2 activity than vipers. Many papers screened the effects of PLA2 isolated from the venoms of different elapids and vipers against different viruses and illustrated the mechanisms of their antiviral effects on different stages of virus replication (Teixeira et al. 2020). Basic sPLA2 purified from the venom of the South American rattlesnake pit viper, *Crotalus durissus terrificus*, inhibited the entry of Dengue virus (DENV), Yellow fever virus (YFV), Hepatitis C virus (HCV), and Chikungunya virus (CHIKV) via direct degradation of viral envelope (Muller et al. 2014; Shimizu et al. 2017; Santos et al. 2021). In addition, Muller et al. (2012) showed that basic PLA2-CB and PLA2-IC from *Crotalus durissus terrificus* had potent in vitro inhibition of YFV and DENV replication. Both proteins displayed high selectivity indices (SI). Similarly, the *Bothrops asper* venom has catalytically active sPLA 2 (Mt-I) and the enzymatically inactive sPLA 2 (Mt-II). However, the Mt-I is markedly more potent than Mt-II isoform (IC 50 of Mt-I and Mt-II were 1.5 and 2768 ng/mL, respectively) against DENV-2 (Brenes et al., 2020). Further, basic sPLA_2_ (CM-II-sPLA_2_) purified from the venom of the Mozambique spitting cobra, *Naja mossambica mossambica*, exhibited higher antiviral activity against HCV, DENV and Japanese encephalitis virus (JEV) (IC50 in ng/ml) than against Sindbis virus (SINV), influenza A virus (IAV), Sendai virus (SV) and Herpes simplex virus (HSV) (IC50 in µg/ml) (Chen et al. 2017). Additionally, potent HIV-1 inhibitors were identified from basic PLA_2_ purified from the venom of three elapids; *Naja mossambica mossambica, Naja nigricollis* and *Oxyuranus scutellatus* (Fenard et al. 1999). Another study by Farzad et al. (2020) demonstrated that PLA_2_ from Iranian Caspian cobra (*Naja Naja Oxiana*) has virucidal activity against the rabies virus. To our knowledge, our study is the first investigation highlighting the virucidal effect of basic *Naja haje haje* PLA2 against rotavirus (RV) and mammalian coronavirus (BCoV).

To get an insight about the lower catalytic activity of basic phospholipase A2 enzymes compared with acidic ones, crystallographic, dynamic light scattering and amino acids comparative structural studies were carried out (dos Santos et al., 2011). The authors indicated that BthTX-II, a basic PLA2 enzyme isolated from the snake *Bothrops jararacussu,* has a distorted calcium-binding loop. This may suggest the low or insignificant catalytic activity of these enzymes. The calcium-binding loop of BthTX-II is composed of Y28, G30, G32 and D49, and the catalytic network contains H48, D49, Y52, and D99. In our *Naja haje* (Egyptian cobra) PLA2, we have the same calcium loop. while the D99 of the catalytic network is absent (data not shown). On the contrary, despite their high catalytic activity, the acidic PLA2 enzymes have less biological effects. Two interesting hypotheses were considered to explain the lack of acidic PLA2 toxicity. The first was offered by Fernandez et al. (2010) who proposed a potential digestive function in *Agkistrodon piscivorus leucostoma* snake venom. The second describes a synergistic action between venom proteins (Resende et al., 2017).

In this context, our aim in this study was to determine the antiviral activities of phospholipase A2 purified from the Egyptian Cobra, *Naja haje haje*, against coronavirus and rotavirus. The cytotoxicity of purified PLA2 was tested in MDBK and MA 104 cell lines prior to the antiviral assay using the MTT assay. This assay is colorimetric and based on the reduction of MTT by the mitochondrial dehydrogenase enzyme of living cells to form a purple formazan that can be measured spectrophotometrically.

**Fig. 4:**
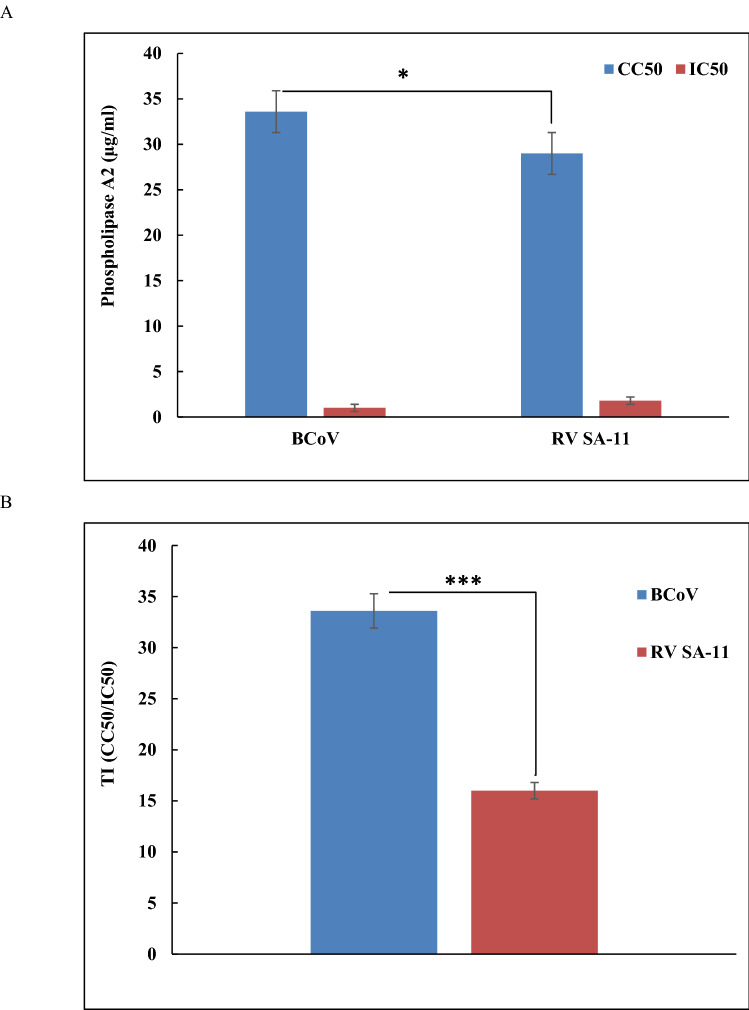
Cytotoxicity and antiviral effects of phospholipase A2 (*N. haje* PLA2) by MTT assay. CC50 is the cytotoxic concentration, IC50 is the half maximal inhibitory concentration and TI is the therapeutic index. Data represent the mean ± SD values (*n* = 3). Comparison test were performed using the GraphPad Prism 8.0 software; *p* < 0.05, *p* < 0.01and *p* < 0.001 are expressed by *, ** and ***, respectively

The values of CC50 and survival rates of *N. haje* PLA2 after incubation with cells were calculated. The PLA2 exhibited low toxicity with high survival rates on MBDK and MA 104, with CC50 of 33.6 and 29 µg/ml, respectively (Fig. 4). Siniavin et al. (2021) showed that the PLA2 isolated from various venoms has low cytotoxicity at 100 μg/ml showed low cytotoxicity on Vero E6 cells. Additionally, the purified *N. haje* PLA2 showed strong antiviral effects against BCoV with a therapeutic index of 33.6 and a reduction in virus titers by 4.25 log_10_TCID_50_/0.1 ml, inhibiting 63% of virus replication. The recent study by Siniavin et al. (2021) showed antiviral activities of five PLA2s against SARS-CoV-2 infections with a potent virucidal activity (100% inhibition) of the phospholipase HDP-2P, isolated from viper *V. nikolskii* venom, at 0.1 μg/ml against SARS-CoV-2 infectivity in Vero E6 cell lines. We would emphasize that the cell types, the nature of each protein and the virus strains may contribute to the different cytotoxic effects.

**Fig. 5:**
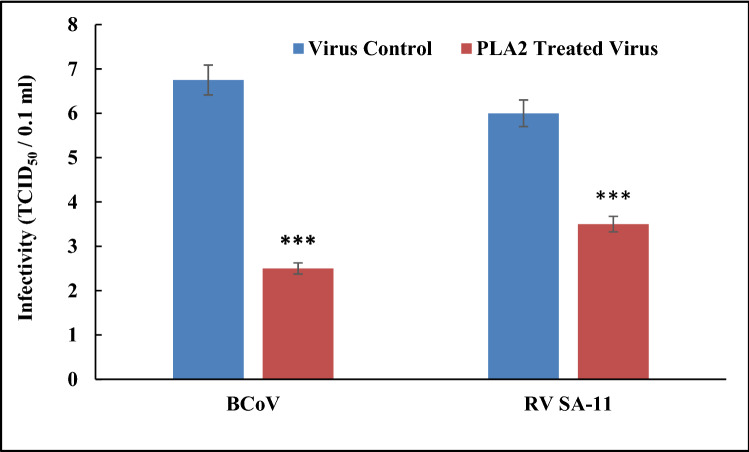
Antiviral effects of phospholipase A2 (*N. haje* PLA2) on BCoV infected MDBK cells and RV infected MA 104 cells by yield reduction assay (TCID_50_/0.1 ml). The *N. haje* PLA2 concentration was used at 3.75 µg/ml. The significance of differences between treated and untreated groups was analyzed by two-sample assuming equal variances *t* test using the GraphPad Prism 8.0 software. Here, *p* < 0.05, *p* < 0.01and *p* < 0.001 are indicated by *, ** and ***, respectively

However, we found lower antiviral activity from the purified PLA2 against RV SA-11 with a TI of 16 and a reduction in virus titers by 2.5 log_10_TCID_50_/0.1 ml, inhibiting 41.7% of virus replication (Figs. 4 and 5).

Several scientists have studied the antiviral activity of PLA2 from the venoms of different species of snakes against several human viruses, including Encephalomyocarditis virus (EMCV), Influenza A (IAV), Coxsackie virus B3 (CVB3), HIV, Dengue virus (DENV), Hepatitis C virus (HCV), Herpes simplex virus (HSV), Japanese encephalitis (JEV), Mayaro virus (MAYV), Middle East respiratory syndrome coronavirus (MERS), SARS-CoV-2 virus, as well as they illustrated the mechanisms of inhibition action against several viruses (Muller et al. 2012, 2014; Cecilio et al. 2013; Russo et al. 2014; Shimizu et al. 2017; Rodrigues et al. 2019; Brenes et al. 2020; Teixeira et al. 2020; Siniavin et al. 2021, 2022; Utkin et al. 2022).

Our antiviral test was based on virucidal activity. Thus, the tested purified protein may inhibit BCoV and RV SA-11 through its interaction with the viral capsid, preventing their attachments to host cells. Siniavin et al. (2021) revealed that *Vipera nikolskii* PLA2 inhibited the SARS-CoV-2 virus in the post-entry stage of virus replication. Additionally, they demonstrated that inhibition of virus was done via either direct hydrolysis of viral phospholipids' envelope or indirectly by inactivating its attachment to virus entry receptor (Angiotensin-converting enzyme 2; ACE2), which spread out in several tissues in the human body, leading to systemic damage. Similarly, other studies demonstrated the pathways where PLA2 interferes with the various stages of virus during replication cycle. sPLA2s have been shown to have the ability to interact with viral capsid (virucidal activity), interfere in viral adsorption (viral entry), interfere in internalization (viral replication), and interfere in host cell components (viral replication) (Muller et al. 2012, 2014; Cecilio et al. 2013; Shimizu et al. 2017; Rodrigues et al. 2019; Russo et al. 2019; Brenes et al. 2020). A limitation of the current study is that other mechanisms of antiviral action (e.g., post-treatment and post-infection) with *N. haje* PLA2 is not included.

## Conclusion

We successfully purified the phospholipase A2 (PLA2) enzyme from the Egyptian cobra *Naja haje haje* venom. The enzyme was suggested to be a basic isoform that mediates toxicological effects. The enzyme showed potential therapeutic indices for both BCoV and RV SA-11 viruses suggesting that *N. haje* PLA2 could successfully demonstrate its potential as an antiviral drug. Further investigations are required to elucidate whether *N. haje* PLA2 inhibits BCoV and RV SA-11 through other mechanisms in vitro and to evaluate the antiviral activity of *N. haje* PLA2 in vivo.

## References

[CR1] Abd El-Aziz MT, Soares AG, Stockand JD (2019). Snake venoms in drug discovery: valuable therapeutic tools for life saving. Toxins.

[CR2] Abid NS, Rouis Z, Lassoued MA, Sfar S, Aouni M (2012). Assessment of the cytotoxic effect and in vitro evaluation of the anti-enteroviral activities of plants rich in flavonoids. J Appl Pharm Sci.

[CR3] Abid I, Jemel I, Alonazi M, Bacha AB (2020). A new group II phospholipase A2 from *Walterinnesiaaegyptia* venom with antimicrobial, antifungal, and cytotoxic potential. Processes.

[CR4] Abidin SAZ, Lee YQ, Othman I, Naidu R (2019). Malaysian cobra venom: a potential source of anti-cancer therapeutic agents. Toxins.

[CR5] Adamude FA, Dingwoke EJ, Abubakar MS, Ibrahim S, Mohamed G, Klein A, Sallau AB (2021). Proteomic analysis of three medically important Nigerian Naja (*Naja haje*, *Naja katiensis* and *Naja nigricollis*) snake venoms. Toxicon.

[CR6] Bradford MM (1976). A rapid and sensitive method for the quantitation of microgram quantities of protein utilizing the principle of protein-dye binding. Anal Biochem.

[CR7] Brahmachari G, Brahmachari G (2011). Natural products in drug discovery: impacts and opportunities—an assessment. Visva-Bharati University, India.

[CR8] Brenes H, Loria GD, Lomonte B (2020). Potent virucidal activity against *Flaviviridae* of a group IIA phospholipase A (2) isolated from the venom of *Bothrops asper*. Biologicals.

[CR9] Calderon LA, Sobrinho JC, Zaqueo KD, de Moura AA, Grabner AN, Mazzi MV, Marcussi S, Nomizo A, Fernandes CF, Zuliani JP, Carvalho BM, da Silva SL, Stábeli RG, Soares AM (2014). Antitumoral activity of snake venom proteins: new trends in cancer therapy. Biomed Res Int.

[CR10] Cecilio AB, Caldas S, Oliveira RA, Santos AS, Richardson M, Naumann GB, Schneider FS, Alvarenga VG, Estevão-Costa MI, Fuly AL, Eble JA, Sanchez EF (2013). Molecular characterization of Lys49 and Asp49 phospholipases A2 from snake venom and their antiviral activities against dengue virus. Toxins.

[CR11] Cedro RCA, Menaldo DL, Costa TR, Zoccal KF, Sartim MA, Santos-Filho NA, Faccioli LH, Sampaio SV (2018). Cytotoxic and inflammatory potential of a phospholipase A_2_ from *Bothropsjararaca* snake venom. J Venom Anim Toxins Incl Trop Dis.

[CR12] Chen M, Aoki-Utsubo C, Kameoka M, Deng L, Terada Y, Kamitani W, Sato K, Koyanagi Y, Hijikata M, Shindo K (2017). Broad-spectrum antiviral agents: secreted phospholipase A2 targets viral envelope lipid bilayers derived from the endoplasmic reticulum membrane. Sci Rep.

[CR13] Costa MIE, Soler RS, Johanningmeier B, Eble JA (2018). Snake venom components in medicine: from the symbolic rod of Asclepius to tangible medical research and application. Int J Biochem Cell Biol.

[CR14] da Mata ÉCG, Mourão CBF, Rangel M, Schwartz EF (2017). Antiviral activity of animal venom peptides and related compounds. J Venom Anim Toxins Incl Trop Dis.

[CR15] Dampalla CS, Zheng J, Perera KD, Wong LR, Meyerholz DK, Nguyen HN, Kashipathy MM, Battaile KP, Lovell S, Kim Y (2021). Post infection treatment with a protease inhibitor increases survival of mice with a fatal SARS-CoV-2 infection. Proc Natl Acad Sci U S A.

[CR16] dos Santos JI, Cintra-Francischinelli M, Borges RJ, Fernandes CA, Pizzo P, Cintra AC, Braz AS, Soares AM, Fontes MR (2011). Structural, functional, and bioinformatics studies reveal a new snake venom homologue phospholipase A2 class. Proteins.

[CR17] El-Aziz TMA, Soares AG, Stockand JD (2019). Snake venoms in drug discovery: valuable therapeutic tools for life saving. Toxins.

[CR18] El-Hakim AE, Gamal-Eldeen AM, Shahein YE, Mansour NM, Wahby AF, Abouelella AM (2011). Purification and characterization of a cytotoxic neurotoxin-like protein from *Najahajehaje* venom that induces mitochondrial apoptosis pathway. Arch Toxicol.

[CR19] Elsayed EA, El-Serehy HA, Salama WH, Al-Misned F (2014). Evaluation of the cytotoxic and antiviral activities of partially purified *Najahajehaje* venom. J Pure Appl Microbiol.

[CR20] Farzad R, Gholami A, Hayati Roodbari N, Shahbazzadeh D (2020). The anti-rabies activity of Caspian cobra venom. Toxicon.

[CR21] Fenard D, Lambeau G, Valentin E, Lefebvre JC, Lazdunski M, Doglio A (1999). Secreted phospholipases A(2), a new class of HIV inhibitors that block virus entry into host cells. J Clin Investig.

[CR22] Fernández J, Gutiérrez JM, Angulo Y, Sanz L, Juárez P, Calvete JJ, Lomonte B (2010). Isolation of an acidic phospholipase A2 from the venom of the snake *Bothrops asper* of Costa Rica: biochemical and toxicological characterization. Biochimie.

[CR23] Filkin SY, Lipkin AV, Fedorov AN (2020). Phospholipase Superfamily: Structure, Functions, and Biotechnological Applications. Biochemistry (mosc).

[CR24] Garfin DE (1990). Isoelectric focusing. Methods Enzymol.

[CR25] Ghosh A, Roy R, Nandi M, Mukhopadhyay A (2019). Scorpion venom-toxins that aid in drug development: a review. Int J Pept Res Ther.

[CR26] Gunst JD, Staerke NB, Pahus MH, Kristensen LH, Bodilsen J, Lohse N, Dalgaard LS, Brønnum D, Fröbert O, Hønge B, Johansen IS, Monrad I, Erikstrup C, Rosendal R, Vilstrup E, Mariager T, Bove DG, Offersen R, Shakar S, Cajander S, Søgaard OS (2021). Efficacy of the TMPRSS2 inhibitor camostat mesilate in patients hospitalized with Covid-19-a double-blind randomized controlled trial. E Clin Med.

[CR27] Gutierrez JM, Avila C, Rojas E, Cerdas L (1988). An alternative in vitro method for testing the potency of the polyvalent antivenom produced in Costa Rica. Toxicon.

[CR28] Hempel BF, Damm M, Petras D, Kazandjian TD, Szentiks CA, Fritsch G, Nebrich G, Casewell NR, Klein O, Süssmuth RD (2022). Spatial venomics—cobra venom system reveals spatial differentiation of snake toxins by mass spectrometry imaging. BioRxiv.

[CR29] Kang TS, Georgieva D, Genov N, Murakami MT, Sinha M, Kumar RP, Kaur P, Kumar S, Dey S, Sharma S, Vrielink A, Betzel C, Takeda S, Arni RK, Singh TP, Kini RM (2011). Enzymatic toxins from snake venom: structural characterization and mechanism of catalysis. FEBS J.

[CR30] Karber G (1931). 50% end-point calculation. Archiv Experiment Pathol Pharmacol.

[CR31] Kaufmann SH, McElrath MJ, Lewis DJ, Del Giudice G (2014). Challenges and responses in human vaccine development. Curr Opin Immunol.

[CR32] Kim CH (2021). Anti–SARS-CoV-2 natural products as potentially therapeutic agents. Front Pharmacol.

[CR33] Koh CY, Kini RM (2012). From snake venom toxins to therapeutics-cardiovascular examples. Toxicon.

[CR34] Laemmli UK (1970). Cleavage of structural proteins during the assembly of the head of bacteriophage T4. Nature.

[CR35] Leung AK, Kellner JD, Davies HD (2005). Rotavirus gastroenteritis. Adv Ther.

[CR36] Malih I, Ahmed Rusmili MR, Tee TY, Saile R, Ghalim N, Othman I (2014). Proteomic analysis of Moroccan cobra *Naja haje legionis* venom using tandem mass spectrometry. J Proteom.

[CR37] Marinetti GV (1965). The action of phospholipase A on lipoproteins. BiochemBiophysActa.

[CR38] Maslow JN (2018). The cost and challenge of vaccine development for emerging and emergent infectious diseases. Lancet Glob Health.

[CR39] Modahl CM, Roointan A, Rogers J, Currier K, Mackessy SP (2020). Interspecific and intraspecific venom enzymatic variation among cobras (*Naja* sp. and *Ophiophagus*
*hannah*). Comp Biochem Physiol C Toxicol Pharmacol.

[CR40] Mohammadi Pour P, Fakhri S, Asgary S, Farzaei MH, Echeverría J (2019). The signaling pathways, and therapeutic targets of antiviral agents: focusing on the antiviral approaches and clinical perspectives of anthocyanins in the management of viral diseases. Front Pharmacol.

[CR41] Moreno E, Alape A, Sanchez M, Gutierrez JM (1988). A new method for the detection of phospholipase A2 variants, identification of isoenzymes in the venoms of new born and adult *Bothrops asper* (Terciopelo) snakes. Toxicon.

[CR42] Muller VD, Russo RR, Cintra AC, Sartim MA, Alves-PaivaRde M, Figueiredo LT, Sampaio SV, Aquino VH (2012). Crotoxin and phospholipases A(2) from *Crotalus durissus terrificus* showed antiviral activity against dengue and yellow fever viruses. Toxicon.

[CR43] Muller VD, Soares RO, dos Santos NN, Trabuco AC, Cintra AC, Figueiredo LT, Caliri A, Sampaio SV, Aquino VH (2014). Phospholipase A2 isolated from the venom of *Crotalus durissus terrificus* inactivates dengue virus and other enveloped viruses by disrupting the viral envelope. PLoS ONE.

[CR44] Rashidi R, Valokola MG, Rad SZK, Etemad L, Roohbakhsh A (2020). Antiplatelet properties of snake venoms: a mini review. Toxin Rev.

[CR45] Resende LM, Almeida JR, Schezaro-Ramos R, Collaço RC, Simioni LR, Ramírez D, González W, Soares AM, Calderon LA, Marangoni S, da Silva SL (2017). Exploring and understanding the functional role, and biochemical and structural characteristics of an acidic phospholipase A 2, AplTx-I, purified from *Agkistrodon piscivorus leucostoma* snake venom. Toxicon.

[CR46] Rivero JVR, de Castro FOF, Stival AS, Magalhães MR, Carmo Filho JR, Pfrimer IAH (2011). Mechanisms of virus resistance and antiviral activity of snake venoms. J Venom Anim Toxins Incl Trop Dis.

[CR47] Rodrigues JP, Azevedo V, Fernanda VP, Zoia MAP, Maia LP, Correia LIV, Costa-Cruz JM, de Melo RV, Goulart LR (2019). The anthelmintic effect on Strongyloidesvenezuelensis induced by BnSP- 6, a Lys49-phospholipase A2 homologue from *Bothrops pauloensis* venom. Curr Top Med Chem.

[CR48] Roy A, Bharadvaja N (2021). Venom-derived bioactive compounds as potential anticancer agents: a review. Int J Pept Res Ther.

[CR49] Russo RR, Müller VDM, Cintra ACO, Figueiredo LTM, Sampaio SV, Aquino VH (2014). Phospholipase A2 crotoxin B isolated from the venom of Crotalus durissus terrificus exert antiviral effect against dengue virus and yellow fever virus through its catalytic activity. J Virol Antivir Res.

[CR50] Russo RR, Dos Santos NN, Cintra ACO, Figueiredo LTM, Sampaio SV, Aquino VH (2019). Expression, Purification and virucidalactivity of two recombinant isoforms of phospholipase A2 from *Crotalusdurissusterrificus*venom. Arch Virol.

[CR51] Salama WH, Ibrahim NM, El Hakim AE, Bassuiny RI, Mohamed MM, Mousa FM, Ali MM (2018). L-Amino acid oxidase from *Cerastesvipera* snake venom: isolation, characterization and biological effects on bacteria and tumor cell lines. Toxicon.

[CR52] Santos IA, Shimizu JF, de Oliveira DM, Martins D, Cardoso-Sousa L, Cintra A, Aquino VH, Sampaio SV, Nicolau-Junior N, Sabino-Silva R, Merits A, Harris M, Jardim A (2021). Chikungunya virus entry is strongly inhibited by phospholipase A2 isolated from the venom of *Crotalus durissus terrificus*. Sci Rep.

[CR53] Shimizu JF, Pereira CM, Bittar C, Batista MN, Campos GRF, da Silva S, Cintra ACO, Zothner C, Harris M, Sampaio SV, Aquino VH, Rahal P, Jardim ACG (2017). Multiple effects of toxins isolated from *Crotalusdurissusterrificus* on the hepatitis C virus life cycle. PLoS ONE.

[CR54] Siniavin AE, Streltsova MA, Nikiforova MA, Kudryavtsev DS, Grinkina SD, Gushchin VA, Mozhaeva VA, Starkov VG, Osipov AV, Lummis S, Tsetlin VI, Utkin YN (2021). Snake venom phospholipase A2s exhibit strong virucidal activity against SARS-CoV-2 and inhibit the viral spike glycoprotein interaction with ACE2. Cell Mol Life Sci.

[CR55] Siniavin A, Grinkina S, Osipov A, Starkov V, Tsetlin V, Utkin Y (2022). Anti-HIV activity of snake venom phospholipase A2s: updates for new enzymes and different virus strains. Int J Mol Sci.

[CR56] Six DA, Dennis EA (2000). The expanding superfamily of phospholipase A2 enzymes: classification and characterization. BiochimBiophysActa.

[CR57] Teixeira SC, Borges BC, Oliveira VQ, Carregosa LS, Bastos LA, Santos IA, Jardim ACG, Freire FM, Martins L, Rodrigues VM, Lopes DS (2020). Insights into the antiviral activity of phospholipases A2 (PLA2s) from snake venoms. Int J Biol Macromol.

[CR58] Towbin H, Staehelin T, Gordon J (1979). Electrophoretic transfer of proteins from polyacrylamide gels to nitrocellulose sheets: procedure and some applications. Proc Natl Acad Sci U S A.

[CR59] Trento MVC, Sales TA, de Abreu TS, Braga MA, Cesar PHS, Marques TR, Marcussi S (2019). Exploring the structural and functional aspects of the phospholipase A_2_ from *Naja* spp. Int J BiolMacromol.

[CR60] Utkin Y, Siniavin A, Kasheverov I, Tsetlin V (2022). Antiviral effects of animal toxins: is there a way to drugs?. Int J Mol Sci.

[CR61] Wahby AF, El Hakim AE, Mahdy EME, Salama WH (2013). Immunochemical studies on phospholipase A2 from *Najanigricollis* venom. Acad J BiologSci (c-PhysiolMol Biol)..

[CR62] Wong KY, Tan KY, Tan NH, Tan CH (2021). A neurotoxic snake venom without phospholipase A2: proteomics and cross-neutralization of the venom from senegalese cobra, *Najasenegalensis* (Subgenus: *Uraeus*). Toxins.

[CR63] Wu F, Zhao S, Yu B, Chen YM, Wang W, Song ZG, Hu Y, Tao ZW, Tian JH, Pei YY, Yuan ML, Zhang YL, Dai FH, Liu Y, Wang QM, Zheng JJ, Xu L, Holmes EC, Zhang YZ (2020). A new coronavirus associated with human respiratory disease in China. Nature.

[CR64] Zhou P, Yang XL, Wang XG, Hu B, Zhang L, Zhang W, Si HR, Zhu Y, Li B, Huang CL, Chen HD, Chen J, Luo Y, Guo H, Jiang RD, Liu MQ, Chen Y, Shen XR, Wang X, Zheng XS, Zhao K, Chen QJ, Deng F, Liu LL, Yan B, Zhan FX, Wang YY, Xiao GF, Shi ZL (2020). A pneumonia outbreak associated with a new coronavirus of probable bat origin. Nature.

